# Exploring the Impact of Social Relationship Modification on Young Female Soccer Players’ Performance in Small-Sided Games

**DOI:** 10.5114/jhk/189425

**Published:** 2024-12-06

**Authors:** Asier Los Arcos, Asier Gonzalez-Artetxe, Sara Lombardero, Oihan Esnal-Arrizabalaga, Jokin Aginaga

**Affiliations:** 1 Department of Physical Education and Sport, Faculty of Education and Sport, University of the Basque Country, UPV/EHU, Vitoria-Gasteiz, Araba, Spain.; 2 Society, Sports, and Physical Exercise Research Group (GIKAFIT), University of the Basque Country, UPV/EHU, Vitoria-Gasteiz, Araba, Spain.; 3 Sociedad Deportiva Eibar, Eibar, Gipuzkoa, Spain.; 4 Institute of Smart Cities (ISC), Public University of Navarre, Iruñea/Pamplona, Navarre, Spain.

**Keywords:** women’s sport, football, training, behavior, time-motion analysis

## Abstract

This study compared young female soccer players’ tactical, conditional, and emotional responses during two small-sided games (SSGs), without restrictions (SSG_free_), and introducing an additional rule (SSG_relationship_: if a player touches an opponent just before she receives the ball, her team wins the ball back with an indirect free kick). Fourteen developmental U14 players participated in two 4 × 6-min seven-a-side games (six each, plus goalkeepers) on a 50-m long × 30-m wide field. Players’ positional data were collected using a GPS to assess their tactical performance (central tendency and entropy measures of the surface area, distance between players and to the nearest opponent, and stretch and spatial exploration indices) and conditional performance (total and low-moderate, high, very high speed, sprinting distance covered, and the number of accelerations and decelerations). Participants also rated their perceived enjoyment and competence using the BECS scale. Tactical central tendency measures were higher during SSG_free_ (p < 0.05) than in SSG_relationship_, but no differences were apparent for entropy and conditional measures (p > 0.05). From bout to bout, central tendency measures of tactical variables decreased more frequently during SSG_free_ than SSG_relationship_. Entropy measures and conditional performance hardly varied between bouts. Enjoyment and perceived competence levels were similar for both SSGs. The findings indicate that modifying the interaction between opponents affects players’ tactical responses more than conditional responses when compared with free play. Specifically, touching an opponent before they receive the ball may encourage players to play closer to their opponents during training tasks.

## Introduction

Besides selecting the potentially best players for their teams, soccer academies have to optimize their training process to ensure the adequate development of the whole player (e.g., tactical, conditional, and emotional dimensions). To this end, playing form activities, those with a game-related focus such as small-sided and conditioned games (SSGs) that imply active decision-making (Ford et al., 2010), are frequently applied in worldwide soccer academies (Güllich, 2019; O’Connor et al., 2018; Partington and Cushion, 2013; Roca and Ford, 2020). Indeed, a newly released study (Emmonds et al., 2023) reported that English Women’s Super League Academy players spent almost 40% of their training time performing SSGs. The extensive assessment of both tactical and conditional responses of young male players during these training drills (Clemente et al., 2022; Hill-Haas et al., 2011; Low et al., 2020; Riboli et al., 2022) has helped coaches to design, select, and plan their training contents during the season. In the same way, assessing perceived enjoyment and competence can be valuable for programming the training intervention based on these pivotal ingredients of motivation (Deci and Ryan, 1985). Unfortunately, despite the call made by Williams and Reilly over two decades ago (2000), few researchers have evaluated female soccer training (Williams et al., 2020).

Academy coaches design training tasks by modifying and combining structural traits according to the response they seek from the players. These modifications may concern one or several features of the training task (Parlebas, 2013): the relationship with the *space* (e.g., field size), with the *time* (e.g., task duration), with the *equipment* (e.g., number of balls), and with *other players* (e.g., how many there are of them). The decision often depends on previous experience on the field or the bench. Thus, empirical evidence regarding female players’ tactical, conditional, and emotional responses can reduce the *distance* between what coaches want (i.e., pursued effects) and what takes place (i.e., obtained effects) during training (Parlebas, 2013). Electronic performance and tracking systems such as global positioning systems (GPSs) make it possible to measure collective behavior and individual conditional efforts during training tasks (Cummins et al., 2013; Rico-González et al., 2020). How players use the space available on the field, the predictability of their movement patterns, and the coordination between them can be assessed using the GPS (Gonzalez-Artetxe and Los Arcos, 2021; Memmert et al., 2017; Rico-González et al., 2021), as well as several external workload variables such as distance covered at different speeds or high-intensity accelerations (Alanen et al., 2023; Asian-Clemente et al., 2022; Chena et al., 2022; Makaruk et al., 2024). Scholars have explored whether modifying the relationship of male soccer players with space, employing alternative field configurations (Coutinho et al., 2019a), eliminating external boundaries (Coutinho et al., 2020) or designating corridors and sectors as spatial references (Coutinho et al., 2019b), restricting the number of touches of the ball (Casamichana et al., 2014), and introducing floaters (Praça et al., 2022) influence their tactical and conditional responses when compared to nonconditioned SSGs. Evaluating the effects of modifying the structural traits of SSGs might enhance female soccer training, but few studies have assessed tactical, conditional, and emotional responses in this population (de Dios-Álvarez et al., 2022; Los Arcos et al., 2023; Ørntoft et al., 2016).

Although manipulating task conditions has been proposed as an appropriate training strategy to lead players’ response (Ometto et al., 2018; Ramos et al., 2020), the way coaches schedule and periodize these tasks and their effects have not been investigated enough in team sports training (Kiely, 2018; Mujika et al., 2018). Given that several repetitions of SSGs are usually performed within the same session, evaluating their impact on the different dimensions of the whole player would complement the assessment of these training forms (Clemente et al., 2022). Knowing how the players’ behavioral response evolves from bout to bout during conditioned or nonconditioned SSGs would help coaches to plan the time devoted to each training content in accordance with their tactical, conditional, and emotional goals. The differences between repetitions found in male soccer players’ external loads (Clemente et al., 2022) drove us to inquire into both tactical and conditional consequences of repeating the same SSG in female academy soccer. Therefore, this study aimed to assess and compare young female soccer players’ tactical, conditional, and emotional responses during repeated free and conditioned SSGs that modified the relationship between opponents.

## Methods

### Participants

Fourteen players from a U14 team (age: 12.3 ± 0.48 years; playing experience: 5.31 ± 1.25 years) of a Women’s Spanish First Division Club (*Liga F*) participated in the study during the competitive period (October 2022). All available players, who had no health issues or injuries, took part in the study. Participants, who were dedicated to soccer as their main sport, had three 75-min sessions per week (on Tuesdays, Thursdays, and Fridays) on an outdoor artificial turf field for 32 weeks during the season. Additionally, they competed in the top U14 league on weekends, finishing the 2022/2023 season in the top half, securing a third-place position out of eight teams. Each participant was considered a trained/developmental player (McKay et al., 2022). Players, their parents/guardians, coaches, and the academy heads were fully informed about the purpose and procedures of the study before giving written informed consent for the children to participate. The study followed the ethical principles for medical research involving human subjects of the World Medical Association (Declaration of Helsinki, 2013) and was approved beforehand by the Ethics Committee for Research involving Human Beings (GIEB in Basque) of the University of the Basque Country (protocol code M10_2021_328; approval date: 25 November 2021).

### Design and Procedures

The study comprised two training sessions performed on nonconsecutive days: Tuesday (MD−4) and Thursday (MD−2). Coaches divided participants into two balanced groups according to the players’ level and position (Gonzalez-Artetxe et al., 2022) following a 1-2-3-1 formation: one goalkeeper, two defenders, three midfielders, and one striker. After a customary 10-min warm-up conducted by the team coach, participants played a 4 × 6-min seven-a-side SSG (six outfield players each, plus goalkeepers) with 3 min of passive rest between bouts in each session. Both SSGs were performed following all official game rules, including the offside rule, and match conditions on a 50-m long × 30-m wide playing field (107 m^2^ × player). Several balls were distributed around the field to ensure fast replacement and to avoid losing time.

With regard to task conditions, one SSG was played freely without additional restrictions (SSG_free_), while the other one included an extra rule: if a player touches an opponent with the hand just before she receives the ball, her team wins the ball back with an indirect free kick (SSG_relationship_). This task condition, which influenced the relationship between opposing players, was novel to the participants. The team coach refereed and penalized any player who broke these rules. Except for this, her intervention was neutralized in order not to influence players’ conduct with her feedback (Gonzalez-Artetxe et al., 2022).

### Measures

Outfield players’ positional data were gathered by a GPS (WIMU PRO, RealTrack Systems, Almeria, Spain) with a 10-Hz sampling frequency to evaluate players’ socio-motor behavior (Bastida-Castillo et al., 2018). Tactical performance comprised central tendency and approximate entropy (ApEn; Pincus, 1991) measures of the surface area (SA [m^2^], calculated by convex hull), distance (m) between players (Dist_players_) and to the nearest opponent (Dist_nearest_), the stretch index (SI, the mean distance [m] of the players to their centroid), and the players’ spatial exploration index (SEI, the distance [m] of each player to her mean position). Conditional performance was assessed by total and low-to-moderate-speed running (lower 2.91 ^m^/_s_), high-speed running (above 2.91 ^m^/_s_), very-high-speed running (above 4.73 ^m^/_s_), and sprinting (above 5.66 ^m^/_s_) distances (m) covered (Harkness-Armstrong et al., 2022); and by the number of accelerations (greater than 2 ^m^/_s_^2^) and decelerations (less than −2 ^m^/_s_^2^) performed (Mara et al., 2015; Silva et al., 2023). All computations were run with MATLAB (version R2020a for Windows, MathWorks, Natick, MA, USA) following existing procedures (Folgado et al., 2014). For ApEn calculation, the length of the compared runs was chosen to be m = 2 and the tolerance factor r = 0.2 × variance of each data set.

Players rated their perceived enjoyment and competence 5 min after the last bout of each SSG using the BECS scale validated by Arias-Estero et al. (2013). This five-point Likert-type scale (1 = *strongly disagree*, 5 = *strongly agree*) has been extensively used within youth team sports (Barquero-Ruiz et al., 2021; Gaztelu-Folla et al., 2022; Morales-Belando et al., 2023; Vélaz-Lorente et al., 2022). Players spent between 3 and 5 min responding to the seven statements of the scale in silence and sitting at least 2 m from each other (so that their peers would not influence their ratings). Four items out of seven (1, 3, 5, and 7) refer to feeling good or considering oneself to be good at playing each SSG, while the remaining items (2, 4, and 6) relate to the contented feeling of each SSG. The average of even and odd items determined perceived enjoyment and competence, respectively.

### Statistical Analysis

Outcomes are presented as means ± standard deviations (SDs). Collective variables (i.e., SA, Dist_players_, Dist_nearest_, and SI central tendency and ApEn measures) were compared between SSG conditions and between bouts of each SSG as “not related” because their values corresponded to the players as a whole, not to a single individual. Thus, values were compared not based on a moment of play (e.g., first second of each SSG), but as a whole (e.g., all data on each SSG or bout). As SEI (the mean value of each player during each SSG or bout) and conditional variables (distances covered and accelerations and decelerations performed during each SSG or bout) were individual variables, these values were compared as “related”: the values of the same player in different SSG conditions or bouts. Mann-Whitney and Kruskal-Wallis (Dwass-Steel-Critchlow-Fligner pairwise comparisons) tests were used to compare collective variables (data were not distributed normally) between SSG_free_ and SSG_relationship_ taking all bouts together, and between bouts within each SSG condition, respectively. An unpaired *t*-test and one-way ANOVA (Tukey post-hoc test) were used to compare the ApEn values of collective variables between SSG_free_ and SSG_relationship_ taking all bouts together, and between bouts within each SSG condition, respectively. Individual SEI, and its ApEn, and conditional variables were compared between SSG conditions taking all bouts together using a paired *t*-test, and between bouts of each SSG condition using repeated measures ANOVA (Tukey post-hoc test). The Wilcoxon signed-rank test and a paired *t*-test were used to compare enjoyment (data were not normally distributed) and perceived competence, respectively. Practical differences were assessed by Cohen’s *d* effect size (thresholds: < 0.20, trivial; 0.20, small; 0.50, moderate; and ≥ 0.80, large) and by Eta squared (η^2^) (thresholds: trivial, < 0.01; small, 0.01–0.06; medium, 0.06–0.14; and large, > 0.14) for parametric and nonparametric comparisons, respectively (Cohen, 1988). The coefficient of variation (CV%) was calculated to assess within-task and inter-player tactical and conditional variabilities, and inter-player perceived enjoyment and competence variabilities. A *p-*value of < 0.05 was considered significant, and all analyses were performed using jamovi software, version 2.3.2.

## Results

Taking the four bouts altogether, SA, Dist_players_, Dist_nearest_, SI, and SEI central tendency measures were significantly higher (*p* < 0.05) during SSG_free_ than in SSG_relationship_, while no significant differences (*p* > 0.05) were found for ApEn measures (Table 1).

**Table 1:** Tactical outcomes for both small-sided games conditions: without restrictions (SSG_free_) and modifying the relationship between opponents (SSG_relationship_).

	SSG_free_	SSG_relationship_	SSG_free_ vs. SSG_relationship_ (Cohen’s *d* or *η*^2^)
**SA (m^2^)**	381 ± 102*	360 ± 106	*η*^2^ = 0.01; trivial
**CV**	26.9%	29.5%	
**ApEn**	0.0969 ± 0.0212	0.0981 ± 0.0183	*d* = 0.06; trivial
**Dist_players_ (m)**	14.3 ± 1.71*	13.9 ± 2.02	*η*^2^ = 0.01; trivial
**CV**	11.9%	14.5%	
**ApEn**	0.1000 ± 0.0229	0.0910 ± 0.0202	*d* = 0.44; small
**Dist_nearest_ (m)**	4.82 ± 1.19*	4.33 ± 1.15	*η*^2^ = 0.05; small
**CV**	24.7%	26.6%	
**ApEn**	0.1890 ± 0.0633	0.1810 ± 0.0586	*d* = 0.17: trivial
**SI (m)**	9.82 ± 1.15*	9.51 ± 1.37	*η*^2^ = 0.01; small
**CV**	11.7%	14.4%	
**ApEn**	0.1070 ± 0.0246	0.0946 ± 0.0230	*d* = 0.51; moderate
**SEI (m)**	8.16 ± 0.92*	7.53 ± 0.88	*d* = 0.70; moderate
**CV**	11.3%	11.7%	
**ApEn**	0.1070 ± 0.0151	0.1100 ± 0.0129	*d* = 0.21; small

Abbreviations: SA: surface area; CV: coefficient of variation; ApEn: approximate entropy; Dist_players_: distance between players; Dist_nearest_: distance to the nearest opponent; SI: stretch index; SEI: spatial exploration index. Note: * significantly higher than SSG_relationship_ (p < 0.05)

Except for distances at low-to-moderate-speed running (*p* < 0.05), significant differences were not apparent (*p* > 0.05) for total and high-speed, very-high-speed, and sprinting distances covered and the accelerations and decelerations performed between SSG conditions (Table 2).

**Table 2:** Conditional outcomes for both small-sided games conditions: without restrictions (SSG_free_) and modifying the relationship between opponents (SSG_relationship_).

	SSG_free_	SSG_relationship_	SSG_free_ vs. SSG_relationship_ (Cohen’s *d*)
**Total distance (m)**	2230 ± 217	2130 ± 195	0.49; small
**CV (%)**	9.7%	9.2%	
**L-M-S running (m)**	1660 ± 72*	1620 ± 73	0.55; moderate
**CV (%)**	4.3%	4.5%	
**H-S running (m)**	508 ± 165	454 ± 139	0.35; small
**CV (%)**	32.5%	30.6%	
**V-H-S running (m)**	49 ± 34	46 ± 36	0.09; trivial
**CV (%)**	68.6%	76.9%	
**Sprinting (m)**	10 ± 10	13 ± 20	0.19; trivial
**CV (%)**	99.3%	155.9%	
**Accelerations > 2** ^m^/_s_^2^	60 ± 15	59 ± 18	0.06; trivial
**CV (%)**	25.0%	30.5%	
**Decelerations < −2** ^m^/_s_^2^	68 ± 18	70 ± 21	0.10; trivial
**CV (%)**	26.5%	30.0%	

Abbreviations: CV: coefficient of variation; L-M-S: low-to-moderate-speed; H-S: high-speed; V-H-S: very-high-speed. Note: * significantly higher than SSG_relationship_ (p < 0.05)

SA, Dist_players_, Dist_nearest_, SI, and SEI central tendency measures were significantly lower during the first bout compared with consecutive bouts of SSG_free_ (Table 3). SA and Dist_nearest_ were also significantly lower from the first to the next bouts during the SSG_relationship_ (Table 4). Entropy differences were not found (*p* > 0.05) during the four bouts of SSG_free_ (Table 3) and SSG_relationship_ (Table 4), except for the ApEn of the SEI during the conditioned SSG (*p* < 0.05; 1^st^ bout higher than the 3^rd^ and the 4^th^).

**Table 3:** Tactical outcomes for small-sided game without restrictions (SSG_free_) bout to bout.

	1^st^ bout	2^nd^ bout	3^rd^ bout	4^th^ bout	Effect sizes (Cohen’s *d* or *η*^2^)
**SA (m^2^)**	421 ± 107^abc^	380 ± 97.8	382 ± 99.1^c^	343 ± 91.4	*η*^2^ 1 vs. 2: 0.04; small / 1 vs. 3: 0.03; small / 1 vs. 4: 0.13; medium / 2 vs. 3: 0.00; trivial / 2 vs. 4: 0.03; small / 3 vs. 4: 0.04; small
**CV**	25.4%	25.7%	25.9%	26.7%	
**ApEn**	0.0905± 0.0107	0.105 ± 0.0211	0.0956 ± 0.0325	0.0965 ± 0.0221	*d* 1 vs. 2: 0.86; large / 1 vs. 3: 0.58; moderate / 1 vs. 4: 0.35; small / 2 vs. 3: 0.34; small / 2 vs. 4: 0.39; small / 3 vs. 4: 0.03; trivial
**Dist_players_ (m)**	15.0 ± 1.77^abc^	14.3 ± 1.64^bc^	14.4 ± 1.44^c^	13.6 ± 1.70	*η*^2^ 1 vs. 2: 0.03; small / 1 vs. 3: 0.02; small / 1 vs. 4: 0.13; medium / 2 vs. 3: 0.00; trivial / 2 vs. 4: 0.04; small / 3 vs. 4: 0.06; medium
**CV**	11.8%	11.5%	10.0%	12.5%	
**ApEn**	0.0905 ± 0.0133	0.1010 ± 0.0158	0.1150 ± 0.0355	0.0948 ± 0.0219	*d* 1 vs. 2: 0.74; moderate / 1 vs. 3: 0.92; large / 1 vs. 4: 0.24; small / 2 vs. 3: 0.50; moderate / 2 vs. 4: 0.34; small / 3 vs. 4: 0.69; moderate
**Dist_nearest_ (m)**	5.30 ± 1.30^abc^	4.67 ± 1.03^bc^	4.94 ± 1.34^c^	4.34 ± 0.82	*η*^2^ 1 vs. 2: 0.06; small / 1 vs. 3: 0.03; small / 1 vs. 4: 0.18; large / 2 vs. 3: 0.00; trivial / 2 vs. 4: 0.03; small / 3 vs. 4: 0.05; small
**CV**	24.5%	22.1%	27.1%	18.9%	
**ApEn**	0.2010 ± 0.0732	0.1770 ± 0.0430	0.1490 ± 0.0521	0.2280 ± 0.0622	*d* 1 vs. 2: 0.41; small / 1 vs. 3: 0.83; large / 1 vs. 4: 0.39; small / 2 vs. 3: 0.82; large / 2 vs. 4: 1.36; large / 3 vs. 4: 1.95; large
**SI (m)**	10.3 ± 1.18^abc^	9.82 ± 1.11^bc^	9.90 ± 0.96^c^	9.33 ± 1.15	*η*^2^ 1 vs. 2: 0.03; small / 1 vs. 3: 0.03; small / 1 vs. 4: 0.14; medium / 2 vs. 3: 0.00; trivial / 2 vs. 4: 0.04; small / 3 vs. 4: 0.07; medium
**CV**	11.5%	11.3%	9.7%	12.3%	
**ApEn**	0.1000 ± 0.0153	0.1030 ± 0.0139	0.1240 ± 0.0376	0.1010 ± 0.0261	*d* 1 vs. 2: 0.15; trivial / 1 vs. 3: 0.81; large / 1 vs. 4: 0.01; trivial / 2 vs. 3: 0.74; moderate / 2 vs. 4: 0.10; trivial / 3 vs. 4: 0.71; moderate
**SEI (m)**	9.49 ± 1.62^abc^	7.97 ± 0.71	7.73 ± 1.19	7.46 ± 0.60	*d* 1 vs. 2: 1.22; large / 1 vs. 3: 1.24; large / 1 vs. 4: 1.66; large / 2 vs. 3: 0.25; small / 2 vs. 4: 0.78; moderate / 3 vs. 4: 0.29; small
**CV**	17.1%	8.9%	15.4%	8.0%	
**ApEn**	0.1090 ± 0.0185	0.1010 ± 0.0190	0.1050 ± 0.0172	0.1140 ± 0.0178	*d* 1 vs. 2: 0.42; small / 1 vs. 3: 0.22; small / 1 vs. 4: 0.28; small / 2 vs. 3: 0.22; small / 2 vs. 4: 0.71; moderate / 3 vs. 4: 0.51; small

Abbreviations: SA: surface area; CV: coefficient of variation; ApEn: approximate entropy; Dist_players_: distance between players; Dist_nearest_: distance to the nearest opponent; SI: stretch index; SEI: spatial exploration index. Note: * ^a^ significantly different (p < 0.05) in comparison to the 2^nd^ bout; ^b^ significantly different (p < 0.05) in comparison to the 3^rd^ bout; ^c^ significantly different (p < 0.05) in comparison to the 4^th^ bout

**Table 4:** Tactical outcomes for small-sided game modifying the relationship between adversaries

	1^st^ bout	2^nd^ bout	3^rd^ bout	4^th^ bout	Effect sizes (Cohen’s *d* or *η*^2^)
**SA (m^2^)**	380 ± 120^abc^	351 ± 96.8	358 ± 93^c^	351 ± 110	*η*^2^ 1 vs. 2: 0.01; small / 1 vs. 3: 0.00; trivial / 1 vs. 4: 0.01; trivial / 2 vs. 3: 0.00; trivial / 2 vs. 4: 0.00; trivial / 3 vs. 4: 0.00; trivial
**CV**	31.6%	27.6%	26.0%	31.3%	
**ApEn**	0.1010 ± 0.0206	0.1020 ± 0.00723	0.1110 ± 0.0195	0.0785 ± 0.00771	*d* 1 vs. 2: 0.07; trivial / 1 vs. 3: 0.50; moderate / 1 vs. 4: 1.48; large / 2 vs. 3: 0.61; moderate / 2 vs. 4: 3.14; large / 3 vs. 4: 2.19; large
**Dist_players_ (m)**	14.1 ± 2.22^ac^	13.9 ± 1.82^b^	14.0 ± 1.73^c^	13.8 ± 2.23	*η*^2^ 1 vs. 2: 0.00; trivial / 1 vs. 3: 0.00; trivial / 1 vs. 4: 0.00; trivial / 2 vs. 3: 0.01; trivial / 2 vs. 4: 0.00; trivial / 3 vs. 4: 0.00; trivial
**CV**	15.8%	13.1%	12.4%	16.2%	
**ApEn**	0.0868 ± 0.0165	0.1020 ± 0.0198	0.1020 ± 0.0202	0.0726 ± 0.0122	*d* 1 vs. 2: 0.83; large / 1 vs. 3: 0.82; large / 1 vs. 4: 0.98; large / 2 vs. 3: 0.00; trivial / 2 vs. 4: 1.79; large / 3 vs. 4: 1.76; large
**Dist_nearest_ (m)**	4.60 ± 1.05^abc^	4.40 ± 1.31^bc^	4.18 ± 0.90^c^	4.13 ± 1.22	*η*^2^ 1 vs. 2: 0.02; small / 1 vs. 3: 0.04; small / 1 vs. 4: 0.05; small / 2 vs. 3: 0.00; trivial / 2 vs. 4: 0.01; trivial / 3 vs. 4: 0.00; trivial
**CV**	22.8%	29.8%	21.5%	29.5%	
**ApEn**	0.1780 ± 0.0548	0.1910 ± 0.0920	0.1940 ± 0.0235	0.1620 ± 0.0497	*d* 1 vs. 2: 0.17; trivial / 1 vs. 3: 0.34; small / 1 vs. 4: 0.31; small / 2 vs. 3: 0.05; trivial / 2 vs. 4: 0.39; small / 3 vs. 4: 0.82; large
**SI (m)**	9.53 ± 1.53	9.55 ± 1.25	9.54 ± 1.18	9.41 ± 1.50	*η*^2^ 1 vs. 2: 0.00; trivial / / 1 vs. 3: 0.00; trivial / 1 vs. 4: 0.00; trivial / 2 vs. 3: 0.00; trivial / 2 vs. 4: 0.00; trivial / 3 vs. 4: 0.00; trivial
**CV**	16.1%	13.1%	12.4%	15.9%	
**ApEn**	0.0899 ± 0.0174	0.1060 ± 0.0242	0.1080 ± 0.0224	0.0749 ± 0.0172	*d* 1 vs. 2: 0.77; moderate / 1 vs. 3: 0.90; large / 1 vs. 4: 0.87; large / 2 vs. 3: 0.09; trivial / 2 vs. 4: 1.48; large / 3 vs. 4: 1.66; large
**SEI (m)**	7.39 ± 0.84^b^	7.16 ± 1.24^b^	8.50 ± 1.07^c^	7.07 ± 0.84	*d* 1 vs. 2: 0.22; small / 1 vs. 3: 1.15; large / 1 vs. 4: 0.38; small / 2 vs. 3: 1.16; large / 2 vs. 4: 0.09; trivial / 3 vs. 4: 1.49; large
**CV**	11.4%	17.3%	12.6%	11.9%	
**ApEn**	0.1220 ± 0.0169^bc^	0.1150 ± 0.0273	0.1040 ± 0.0130	0.0989 ± 0.0167	*d* 1 vs. 2: 0.31; small / 1 vs. 3: 1.19; large / 1 vs. 4: 1.38; large / 2 vs. 3: 0.51; moderate / 2 vs. 4: 0.71; moderate / 3 vs. 4: 0.34; small

Abbreviations: SA: surface area; CV: coefficient of variation; ApEn: approximate entropy; Dist_players_: distance between players; Dist_nearest_: distance to the nearest opponet; SI: stretch index; SEI: spatial exploration index. Note: * ^a^ significantly different (p < 0.05) in comparison to the 2^nd^ bout; ^b^ significantly different (p < 0.05) in comparison to the 3^rd^ bout; ^c^ significantly different (p < 0.05) in comparison to the 4^th^ bout

Significant differences were not apparent (*p* > 0.05) for conditional performance between bouts of both SSG conditions, free (Table 5) and modifying the relationship among opponents (Table 6), except for six pairwise comparisons (SSG_free_: L-M S running, 1^st^ vs. 2^nd^ and 2^nd^ vs. 3^rd^ bouts; H S running, 1^st^ vs. 4^th^ bouts; decelerations, 1^st^ vs. 3^rd^ and 1^st^ vs. 4^th^; SSG_relationship_: L-M S running, 2^nd^ vs. 3^rd^ bouts).

**Table 5:** Conditional outcomes for small-sided game without restrictions (SSG_free_) bout to bout.

	1^st^ bout	2^nd^ bout	3^rd^ bout	4^th^ bout	Effect sizes (Cohen’s *d*)
**Total distance (m)**	572 ± 75	561 ± 48	557 ± 69	541 ± 47	1 vs. 2: 0.20; small / 1 vs. 3: 0.21; small / 1 vs. 4: 0.50; moderate
**CV (%)**	13.1%	8.6%	12.4%	8.7%	2 vs. 3: 0.07; trivial / 2 vs. 4: 0.42; small / 3 vs. 4: 0.27; small
**L-M-S running (m)**	399 ± 26	432 ± 26^a^	409 ± 25	423 ± 18	1 vs. 2: 1.29; large / 1 vs. 3: 0.40; small / 1 vs. 4: 1.11; large
**CV (%)**	6.4%	5.9%	6.1%	4.2%	2 vs. 3: 0.91; large / 2 vs. 4: 0.41; small / 3 vs. 4: 0.65; moderate
**H-S running (m)**	153 ± 69^b^	118 ± 42	133 ± 53	105 ± 27	1 vs. 2: 0.61; moderate / 1 vs. 3: 0.32; small / 1 vs. 4: 0.92; large
**CV (%)**	45.4%	35.4%	40.1%	32.5%	2 vs. 3: 0.31; small / 2 vs. 4: 0.40; small / 3 vs. 4: 0.67; moderate
**V-H-S running (m)**	17 ± 13	10 ± 9	12 ± 14	10 ± 10	1 vs. 2: 0.67; moderate / 1 vs. 3: 0.36; small / 1 vs. 4: 0.09; trivial
**CV (%)**	76.6%	95.1%	112.2%	96.0%	2 vs. 3: 0.23; small / 2 vs. 4: 0.01; trivial / 3 vs. 4: 0.03; trivial
**Sprinting (m)**	3 ± 5	1 ± 2	3 ± 6	3 ± 3	1 vs. 2: 0.59; moderate / 1 vs. 3: 0.14; trivial / 1 vs. 4: 0.22; small
**CV (%)**	138.9%	170.5%	211.3%	111.6%	2 vs. 3: 0.34; small / 2 vs. 4: 0.52; moderate / 3 vs. 4: 0.03; trivial
**Accelerations > 2** ^m^/_s_^2^	16 ± 5	15 ± 3	15 ± 6	14 ± 4	1 vs. 2: 0.24; small / 1 vs. 3: 0.18; trivial / 1 vs. 4: 0.44; small
**CV (%)**	31.3%	20.0%	40.0%	28.6%	2 vs. 3: 0.00; trivial / 2 vs. 4: 0.28; small / 3 vs. 4: 0.20; small
**Decelerations < −2** ^m^/_s_^2^	21 ± 6^c^	18 ± 6	15 ± 6	16 ± 5	1 vs. 2: 0.50; moderate / 1 vs. 3: 1.00; large / 1 vs. 4: 0.91; large
**CV (%)**	28.6%	33.3%	40.0%	31.3%	2 vs. 3: 0.50; moderate / 2 vs. 4: 0.36; small / 3 vs. 4: 0.18; trivial

Abbreviations: CV: coefficient of variation; L-M-S: low-to-moderate-speed; H-S: high-speed; V-H-S: very-high-speed. Note: ^a^ significantly (p < 0.05) higher than the 1^st^ and the 3^rd^ bout; ^b^ significantly (p < 0.05) higher than the 4^th^ bout; ^c^ significantly (p < 0.05) higher than the 3^rd^ and the 4^th^ bout

**Table 6:** Conditional outcomes for small-sided game modifying the relationship between opponents (SSG_relationship_) bout to bout.

	1^st^ bout	2^nd^ bout	3^rd^ bout	4^th^ bout	Effect sizes (Cohen’s *d*)
**Total distance (m)**	525 ± 54	554 ± 64	531 ± 51	521 ± 44	1 vs. 2: 0.49; small / 1 vs. 3: 0.1; trivial / 1 vs. 4: 0.08; trivial
**CV (%)**	10.3%	11.6%	9.6%	8.5%	2 vs. 3: 0.40; small / 2 vs. 4: 0.60; moderate / 3 vs. 4: 0.21; small
**L-M-S running (m)**	405 ± 17	413 ± 22*	398 ± 25	402 ± 23	1 vs. 2: 0.41; small / 1 vs. 3: 0.33; small / 1 vs. 4: 0.15; trivial
**CV (%)**	4.2%	5.3%	6.3%	5.7%	2 vs. 3: 0.64; moderate / 2 vs. 4: 0.49; small / 3 vs. 4: 0.17; trivial
**H-S running (m)**	108 ± 41	127 ± 47	116 ± 37	103 ± 34	1 vs. 2: 0.43; small / 1 vs. 3: 0.21; small / 1 vs. 4: 0.13; trivial
**CV (%)**	38.0%	37.0%	31.9%	33.0%	2 vs. 3: 0.26; small / 2 vs. 4: 0.59; moderate / 3 vs. 4: 0.37; small
**V-H-S running (m)**	9 ± 7	12 ± 12	11 ± 10	14 ± 12	1 vs. 2: 0.31; small / 1 vs. 3: 0.23; small / 1 vs. 4: 0.51; moderate
**CV (%)**	77.8%	100.0%	90.9%	85.7%	2 vs. 3: 0.09; trivial / 2 vs. 4: 0.17; trivial / 3 vs. 4: 0.27; small
**Sprinting (m)**	2 ± 3	2 ± 4	6 ± 11	3 ± 6	1 vs. 2: 0.00; trivial / 1 vs. 3: 0.50; moderate / 1 vs. 4: 0.21; small
**CV (%)**	150.0%	200.0%	183.3%	200.0%	2 vs. 3: 0.48; small / 2 vs. 4: 0.20; small / 3 vs. 4: 0.34; small
**Accelerations > 2** ^m^/_s_^2^	14 ± 4	14 ± 5	15 ± 7	16 ± 5	1 vs. 2: 0.00; trivial / 1 vs. 3: 0.18; trivial / 1 vs. 4: 0.44; small
**CV (%)**	28.6%	35.7%	46.7%	31.3%	2 vs. 3: 0.16; trivial / 2 vs. 4: 0.40; small / 3 vs. 4: 0.16; trivial
**Decelerations < −2** ^m^/_s_^2^	17 ± 5	18 ± 6	16 ± 6	16 ± 4	1 vs. 2: 0.18; trivial / 1 vs. 3: 0.18; trivial / 1 vs. 4: 0.22; small
**CV (%)**	29.4%	33.3%	37.5%	25.0%	2 vs. 3: 0.33; small / 2 vs. 4: 0.39; small / 3 vs. 4: 0.00; trivial

Abbreviations: CV: coefficient of variation; L-M-S: low-to-moderate-speed; H-S: high-speed; V-H-S: very-high-speed. Note: * significantly (p < 0.05) higher than the 1^st^ bout

No significant differences (*p* > 0.05) between the two SSG conditions were apparent in players’ perceived enjoyment (SSG_free_, 4.81 ± 0.26 vs. SSG_relationship_, 4.71 ± 0.48; η^2^ = 0.02, trivial) and perceived competence (SSG_free_, 3.77 ± 0.53 vs. SSG_relationship_, 3.76 ± 0.41; Cohen’s *d* = 0.02, trivial) values. Enjoyment variabilities between players were 5.4% and 10.2% for SSG_free_ and SSG_relationship_, respectively. Inter-player variabilities for perceived competence were 14.1% and 10.9% for SSG_free_ and SSG_relationship_, respectively.

## Discussion

This study assessed and compared young female soccer players’ tactical, conditional, and emotional responses during repeated free and conditioned SSGs. After modifying the social interaction between opponents, players occupied and explored less space and played closer to each other, but the predictability of tactical performance did not vary in comparison to SSG_free_. In addition, conditional performance hardly varied, and enjoyment and perceived competence levels were very similar between both task conditions. Differences between bouts were more for tactical central tendency measures during SSG_free_ in comparison to SSG_relationship_, but entropy values and conditional performance hardly varied between bouts under both SSG conditions. The findings indicate that modifying the interaction between opponents affects players’ tactical responses more than conditional responses (compared with free play). In particular, touching opponents before receiving the ball can acclimatize players to moving closer to their opponents during training tasks. Additionally, both SSG conditions cause players to behave similarly during several bouts of the same training session.

As with previous studies of male soccer players (Casamichana et al., 2014; Coutinho et al., 2019a, 2019b, 2020; Praça et al., 2022), the present study may provide soccer academy coaches with practical insights into the impact of modifying the structural traits of the task on female players’ overall response (i.e., considering all the players together and without differentiating between phases of the play). In the present case, touching an opponent before receiving the ball implied that the ball was going to be won back, thus players were nearer to each other, and occupied and explored less space during the conditioned SSG than in the free one (Table 1). Just as coaches design and implement activities to induce their players to acquire and practice particular collective patterns and individual habits that enhance their socio-motor competence (Gréhaigne and Godbout, 1995; Newell and Rovegno, 2021), modifying the relationship with their opponents may encourage them to play closer together; for example, touching an opponent before receiving the ball may familiarize players with playing closer to their opponents during training tasks.

A similar study found that U12 female soccer players explored more space (*p* < 0.05) during non-conditioned SSGs than in SSGs with obstacles (i.e., cones, mini-goals, and saucer cones) scattered across the field, whereas differences were not found between task conditions for Dist_players_ and SI variables (Los Arcos et al., 2023). For coaches aiming to incite players to explore space, free play may prove more effective than conditioned tasks. It has been suggested that altering task conditions by introducing extra rules or placing obstacles on the field may stress and perturb players’ responses and boost their functionality in uncertain training scenarios (Santos et al., 2018; Schöllhorn et al., 2012). However, no significant differences (*p* < 0.05) were found between SSG conditions for ApEn values in all tactical variables (Table 1). Similarly, Dist_players_ and SI entropies were slightly higher (*p* < 0.05; Cohen’s *d* = small) during free play than with field obstacles (Los Arcos et al., 2023). Coaches who want to raise young female players’ levels of unpredictability and make them more adaptable in training and competitive settings replete with affordances should therefore modify task conditions more radically or exaggeratedly (Santos et al., 2018; Schöllhorn et al., 2012).

Among the few studies that have evaluated conditional performance in young female soccer players (de Dios-Álvarez et al., 2022), Ørntoft et al. (2016) modified the relation *with others* during training tasks to compare total distances and distances covered at different speed running intervals in U11 female soccer players. This study altered the number of people (7 vs. 7 and 8 vs. 8 plus goalkeepers), but not the interaction among players, such as the counter-communications among opponents (Parlebas, 2013). After this modification, players’ conditional performance hardly varied between SSG conditions (Table 2). In contrast to the modification of the number of players involved (Ørntoft et al., 2016), distances covered at low-to-moderate-speed running were moderately higher during non-conditioned SSGs (i.e., SSG_free_) than in SSG_relationship_, but differences were not apparent at higher intensities and during accelerations and decelerations. It appears that modifying the interaction between defenders and attackers using an additional rule barely impacts young female players’ conditional dimension when compared with SSG_free_.

Tactical central tendency measures decreased from the beginning to the final bout of SSG_free_, suggesting that players need several repetitions to adjust their tactical performance. However, this tendency was not so clear in SSG_relationship_: the changes between bouts were less frequent, and their magnitudes ranged from trivial to small. Thus, conditioning the relationship between opponents may therefore encourage players to behave similarly across several bouts, and this may help to construct a relatively stable playing style (Gréhaigne and Godbout, 1995). Entropy measures were also similar during bouts of both SSG conditions; SSG_relationship_ does not appear to enrich task complexity (Pincus, 1991), at least not enough to alter players’ tactical response compared with free play. The little conditional variation between bouts (Tables 5 and 6) also suggests that this modification does not considerably affect players’ conditional performance. Thus, female soccer academy coaches can ensure a similar and stable average conditional response from their teams implementing 4 × 6-min seven-a-side SSGs with 3 min of passive rest between bouts within the session. Nevertheless, as previous studies had found (Ørntoft et al., 2016), they should expect high inter-player conditional variability (Tables 5 and 6). Training load quantification at the individual level is necessary to adapt the training contents to certain players if necessary.

No significant differences were found in U14 female players’ perceived enjoyment and competence levels between SSG conditions. Similarly, significant differences were not apparent when U12s played SSGs freely and with field obstacles (Los Arcos et al., 2023). These results suggest that modifying the structural traits of the game during SSGs does not mean that players enjoy it less or perceive themselves as less competent. Perceived enjoyment and competence levels attained playing freely (enjoyment: 4.81 ± 0.26; competence: 3.77 ± 0.53) or altering the relationship between opponents (enjoyment: 4.71 ± 0.48; competence: 3.76 ± 0.41) were similar and considerably high. U12 female soccer players also declared high levels for free (enjoyment: 4.15 ± 0.63; perceived competence: 3.63 ± 1.13) and with-obstacles (enjoyment: 3.42 ± 0.97; perceived competence: 3.33 ± 0.98) SSGs (Los Arcos et al., 2023). In comparison to this study, perceived enjoyment (SSG_free_, CV = 15.2%; SSG_obstacles_, CV = 31.1%) and competence (SSG_free_, CV = 28.4%; SSG_obstacles_, CV = 29.4%) inter-player variabilities were considerably greater in the youngest (i.e., U12) players (Los Arcos et al., 2023). Further studies should assess whether the greater variability values of the previous study were due to the players’ age (U12 vs. U14) or the task condition implemented.

## Conclusions

The scope of the present study was limited in terms of the sample (a single team) and the length of the intervention (two training sessions, one for each SSG), thus the results could not be generalized. Despite its limitations, the study adds to our understanding of academy women’s soccer training, a subject that has been underresearched (particularly from a holistic perspective). By altering the relationship among players, in the present instance with the opponent, coaches might encourage their players to play closer without affecting their conditional performance, unpredictability or perceived enjoyment and competence. Further research with more training sessions and including more academy teams should be undertaken to explore how modifying soccer internal logic within SSGs impacts young female players’ tactical and conditional performance and their emotional experience.
